# Induction of neutralizing antibody response against koala retrovirus (KoRV) and reduction in viral load in koalas following vaccination with recombinant KoRV envelope protein

**DOI:** 10.1038/s41541-018-0066-4

**Published:** 2018-08-02

**Authors:** O Olagoke, D Miller, F Hemmatzadeh, T Stephenson, J Fabijan, P Hutt, S Finch, N Speight, P Timms

**Affiliations:** 1 Genecology Research Center, Faculty of Science, Health, Education and Engineering, University of the Sunshine Coast, 90 Sippy Downs Drive, Sippy Downs, QLD 4556 Australia; 2 School of Animal and Veterinary Sciences, The University of Adelaide, Roseworthy, SA 5371 Australia; 3 The Adelaide Koala and Wildlife Hospital, Plympton, SA 5038 Australia

## Abstract

Koala retrovirus (KoRV) infects the majority of Australia’s koalas (*Phascolarctos cinereus*) and has been linked to several life-threatening diseases such as lymphoma and leukemia, as well as *Chlamydia* and thus poses a threat to the continued survival of this species. While quarantine and antiretroviral drug treatment are possible control measures, they are impractical, leaving vaccination as the only realistic option. In this study, we examined the effect of a recombinant envelope protein-based anti-KoRV vaccine in two groups of South Australian koalas: KoRV infected or KoRV free. We report a successful vaccination response in the koalas with no vaccine-associated side effects. The vaccine induced a significant humoral immune response as well as the production of neutralizing antibodies in both groups of koalas. We also identified B-cell epitopes that were differentially recognized in KoRV-infected versus KoRV-free koalas following vaccination. Importantly, we also showed that vaccination had a therapeutic effect on koalas infected exogenously with KoRV by reducing their circulating viral load. Together, this study highlights the possibility of successfully developing a vaccine against KoRV infection in koalas.

## Koala retrovirus: Hopes for an effective vaccine

A vaccine candidate for Koala retrovirus elicits a protective antibody response and reduces the viral load in already-infected koalas. Koala retrovirus (KoRV), first identified in the last 20 years, is a life-threatening, endemic pathogen affecting Australian koalas. In pursuit of an effective KoRV vaccine, the University of the Sunshine Coast’s Peter Timms led a group of Australian scientists to develop a candidate based on the transmembrane section of the virus’ envelope protein. The six koalas vaccinated in the study all generated a strong antibody response to the envelope protein, and a strong neutralizing antibody response was reported during in vitro tests. Vaccinated koalas with pre-existing KoRV infection benefited from an average 79% reduction in viral load when measured 12 weeks after vaccination. Further research should be prioritized to provide much-needed protection to Australia’s koalas.

## Introduction

Koala retrovirus (KoRV) is a gammaretrovirus that was identified as recently as just two decades ago,^1^ and appears to be spreading through the Australian koala population, from north to south, with 100% of northern Australian koalas infected but less than 50% of southern animals currently infected.^2^ KoRV is currently undergoing endogenization into the koala genome, and as such is the only retrovirus currently known to be doing so.^3^ It is thought that northern koalas carry both the endogenous and exogenous variants of KoRV, while KoRV is present in the exogenous form in southern koalas.^2^ The existence of KoRV in both endogenous and exogenous states means that it is capable of being transmitted both vertically (parent to offspring through the germ line) and horizontally (between infected animals). There are two main variants of KoRV currently recognized, KoRV-A and KoRV-B. Whereas KoRV-A has been shown to exist in both exogenous and endogenous states, KoRV-B is believed to currently exist only in the exogenous state, and is found only among northern koalas. In addition to these two well-described KoRV variants, other variants, KoRV-C to KoRV-I, have also been described.^2,4,5^ KoRV-related lymphomas have been reported in several captive populations^6,7^ and, while not as prevalent, also occur in wild koala populations.^8,9^ KoRV has also been shown to be associated with chlamydial disease in wild koalas.^8,10^ While quarantine and antiretroviral drug treatment are possible control measures, they are impractical, leaving the development of an effective KoRV vaccine as the most realistic option to reduce the threat posed by KoRV in Australian koalas.

Human as well as animal hosts have previously been shown to produce immune responses following natural presentation of retroviral antigens,^11–14^ although not all of these antibodies are protective against the establishment of infection. In cases where natural antibodies are not protective, vaccines may be used to induce the production of protective immunity, with Feline Leukemia Virus (FeLV) representing a classic example. Recombinant envelope protein-based vaccines are designed to induce antibody production against epitopes on the envelope of the retroviruses, and have been shown to induce not only binding antibodies but also neutralizing antibodies, in different viral infection models.^15–17^ Retroviruses are known to replicate by inserting their genome into the host genome, and KoRV, in particular, has been shown to have multiple insertion sites of up to 133 in one investigated koala, with most integrations containing full-length provirus.^18^ Upon integration, retroviruses use the host’s machinery to make copies of themselves. Hence, the induction of neutralizing antibodies becomes important in preventing virus entry into the target cell, and thereby preventing infection. Feibig et al.^19^ showed that rats and goats can be induced to produce KoRV-neutralizing antibodies when vaccinated with KoRV envelope protein, and hypothesized that the KoRV envelope protein may be a suitable vaccine candidate to protect uninfected koalas against KoRV infection. A critical requirement of a vaccine, besides protecting against infection in uninfected animals, is whether it can reduce the viral load in infected animals. As such, therapeutic vaccines represent a promising approach in the effort to enhance immunogenicity, reduce bacterial and viral load in both human and animal infections, and reduce pathogen associated morbidity and mortality.^20,21^

In this study, we investigated the possibility of designing a vaccine that could prevent KoRV infection as well as clear existing infections in koalas. To do this, we evaluated the potential of the transmembrane domain of KoRV envelope protein as a suitable vaccine candidate against KoRV infection in both exogenously infected and uninfected South Australian koalas. We showed that vaccination can be successful for both KoRV-negative and KoRV-positive koalas. All vaccinated animals produced strong levels of anti-KoRV antibodies. We also showed that these antibodies had a neutralizing effect in an in vitro assay, and most importantly, we showed that vaccination had a therapeutic effect on koalas infected with KoRV by reducing the circulating viral load. We also identified B-cell epitopes that were differentially recognized in KoRV-infected or KoRV-free koalas following vaccination.

## Results

### KoRV status of trial animals

Using the *pol* gene PCR assay, three of six koalas (Kp1, Kp2, and Kp3) tested positive for KoRV infection. Genotyping of the KoRV infection showed that these three koalas were positive for KoRV-A but negative for KoRV-B. These three KoRV PCR positive koalas were determined to be infected with exogenous KoRV-A judging by the estimated proviral copy number of less than one copy per cell. The other three koalas (Kn4, Kn5, and Kn6) tested negative for KoRV (Table 1). None of the koalas in this trial tested positive for KoRV-B.

**Table 1:** KoRV infection status of trial koalas confirmed by PCR assays

Koala ID	KoRV infection status (by *pol* gene PCR)	*Env* gene PCR assay	
		KoRV-A	KoRV-B
Kp1	Positive	Positive	Negative
Kp2	Positive	Positive	Negative
Kp3	Positive	Positive	Negative
Kn4	Negative	Negative	Negative
Kn5	Negative	Negative	Negative
Kn6	Negative	Negative	Negative

### KoRV antibody status of all koalas, prior to vaccination

**Fig. 1:**
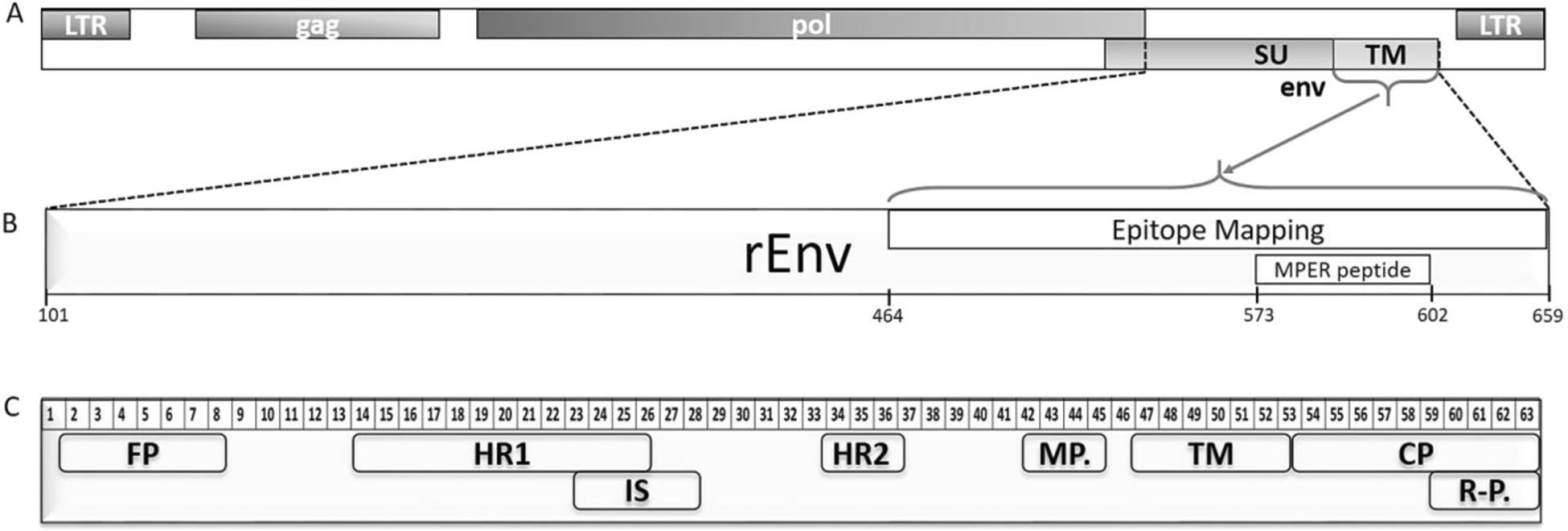
Schematics showing KoRV genome and segment of env protein used for vaccination. **a** Representative KoRV genome showing coding regions. **b** Schematic showing segment of env protein used for vaccination, epitope mapping analysis, and membrane proximal external region (MPER) peptide synthesis. rEnv: recombinant envelope protein used for vaccination. The numbers shown represent corresponding amino acid number (GenBank: AF151794). **c** Layout of the transmembrane subunit of KoRV envelope protein showing important regions. The numbers represent 15mer amino acids peptides overlapping by 12 amino acids. FP fusion peptide, HR1 heptad repeat 1, IS immunosuppressive domain, HR2 heptad repeat 2, MP membrane proximal external region, TM transmembrane region, CP cytoplasmic tail, R-P R-peptide

**Fig. 2:**
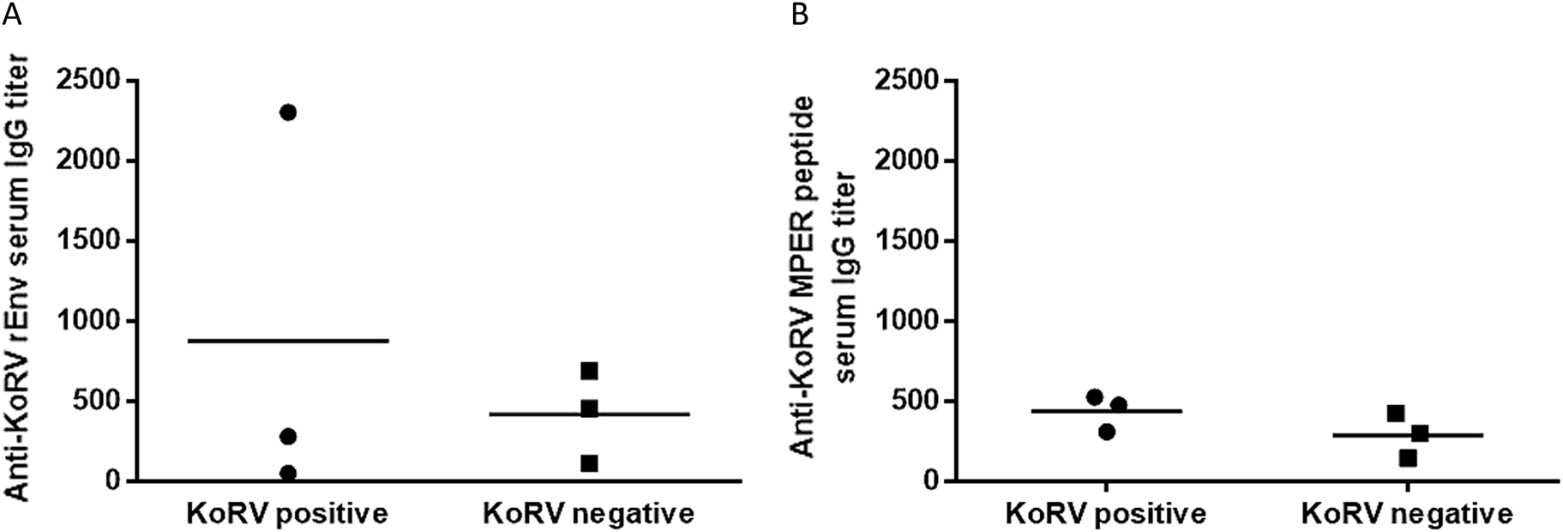
KoRV antibody status pre-vaccination. **a** Anti-KoRV rEnv serum IgG. **b** Anti-KoRV MPER Peptide serum IgG response in KoRV-positive (*n* = 3) and KoRV-negative (*n* = 3) animals prior to vaccination. Horizontal bars represent mean end-point titers of serum IgG levels of the three koalas in each group

As there are very little existing data on the immune response in koalas with exogenous KoRV infection, all trial koalas were characterized for the presence of anti-KoRV antibody prior to vaccination. We developed two in-house ELISAs using recombinant KoRV envelope protein (rEnv) and a synthetic peptide spanning the membrane proximal external region (MPER) of the KoRV envelope protein as antigens (Fig. 1a and b), and used these assays to evaluate the natural level of serum circulating antibodies to KoRV in all six koalas. Our results (Fig. 2) show that while one KoRV-positive koala had a medium-high anti-KoRV rEnv serum IgG titer, the other two KoRV-positive koalas did not have any natural anti-KoRV antibodies All three animals were negative for antibodies against the synthetic MPER peptide.

### Vaccine safety

All six vaccinated koalas, aged around 2 years, were monitored daily throughout the 12-week duration of the trial, with regular health assessments carried out by veterinarians. No vaccine-related adverse reactions were observed in any of the koalas, confirming the safety of the vaccine.

### Vaccinated koalas develop a strong anti-KoRV antibody response

**Fig. 3:**
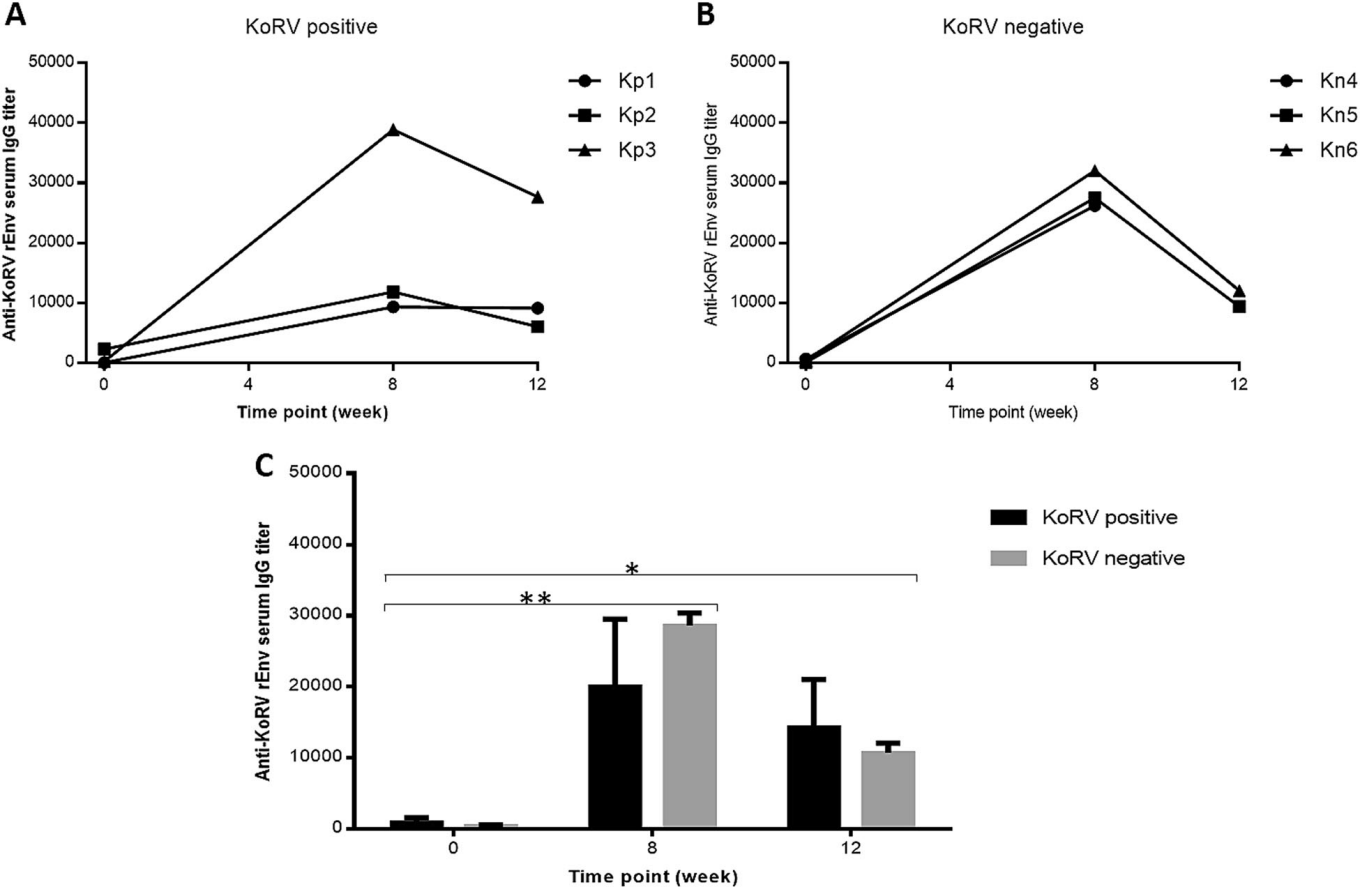
Antibody (IgG) levels expressed as end-point titers in vaccinated KoRV-positive **a** and vaccinated KoRV-negative **b** animals at 0, 8, and 12 weeks post-vaccination. **c** The average serum IgG titers in KoRV-positive and KoRV-negative vaccinated koalas at 0, 8, and 12 weeks post-vaccination were compared and presented as the mean ± SEM of three koalas in KoRV-positive or KoRV-negative group. The level of significance was measured as *p = 0.0183, **p = 0.0012 using Student’s *T* test (*p* ≤ 0.05)

Six koalas were divided into two groups (*n* = 3 per group) based on their KoRV status as determined by KoRV PCR. The animals were vaccinated subcutaneously on day zero and then received a booster dose at 4 weeks. Each vaccine dose contained 50 µg of rEnv protein, with 250 µg each of PCEP, poly I:C, and 500 µg of peptide 1002. Using our rEnv ELISA to quantify serum IgG response at time points 0, 8, and 12 weeks post-vaccination, we observed the production of strong anti-rEnv antibody levels in all koalas at 8 and 12 weeks post-vaccination. One KoRV-positive koala had a particularly high antibody level at weeks 8 and 12, compared to the other two KoRV-positive koalas (Fig. 3a). All KoRV-negative koalas had comparable antibody responses at each time point post-vaccination (Fig. 3b). The average serum IgG titers in KoRV-positive and KoRV-negative vaccinated koalas at 0, 8, and 12 weeks post-vaccination were compared using a two-way ANOVA. The analysis showed that whereas there was no difference in antibody titers based on KoRV status, post-vaccination antibody titers were significantly increased at weeks 8 (*p* = 0.0012) and 12 (*p* = 0.0183) compared to week 0 (Fig. 3c).

### Vaccine-induced production of serum-neutralizing antibodies

**Fig. 4:**
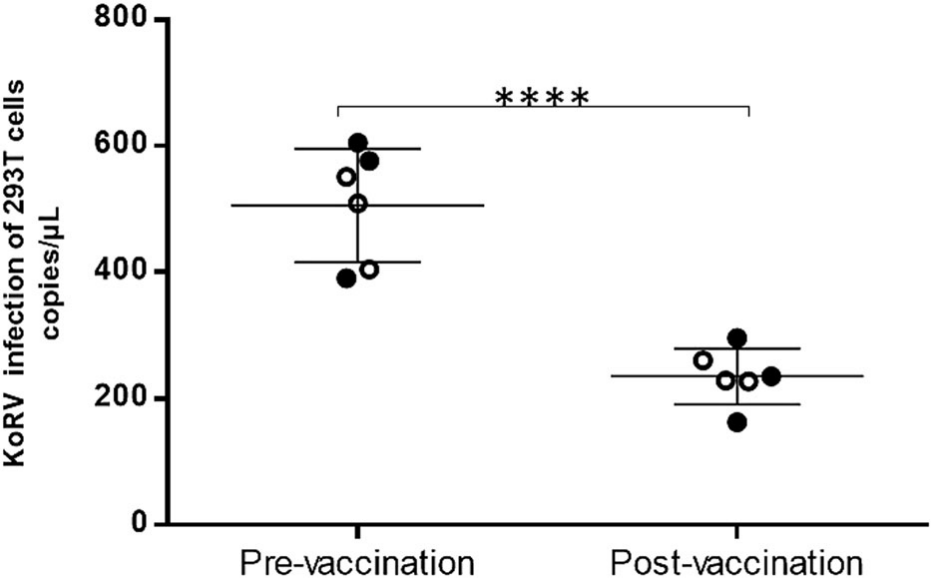
Vaccine-induced neutralizing activity of koala serum as measured by KoRV infection of 293T cells in the presence of koala sera. All post-vaccination serum samples (*n* = 6) were assayed and compared to pre-vaccination samples (mean values and standard deviation are indicated by horizontal lines and error bars, respectively). KoRV-negative samples are shown in open circles while KoRV-positive samples are shown in shaded circles. The level of significance was measured as ****p < 0.0001 using Student’s *T* test (*p* ≤ 0.05)

The production of serum neutralizing antibodies following vaccination was investigated by infecting human embryonic kidney (HEK) 293T cells with KoRV in the presence of pre- or post-vaccination serum. Genomic DNA from infected HEK293T cells was extracted after 65 h of infection and analyzed for KoRV proviral integration. The integration of KoRV into HEK293T cells was compared between pre-vaccination and post-vaccination sera. The presence of neutralizing antibodies is defined as a significant reduction in KoRV infection load in HEK293T cells in the presence of post-vaccination sera. Post-vaccination sera from all six vaccinated koalas were able to significantly neutralize KoRV infection in vitro using a 1:20 serum dilution (Fig. 4). The level of significance was measured as *p < 0.0001 using Student’s *T* test (*p* ≤ 0.05). The reduction in KoRV infectivity of the 293T cells in the presence of post-vaccination serum for individual koala ranged from 2- to 3-fold reduction.

### Reduced KoRV viral RNA load following vaccination

**Fig. 5:**
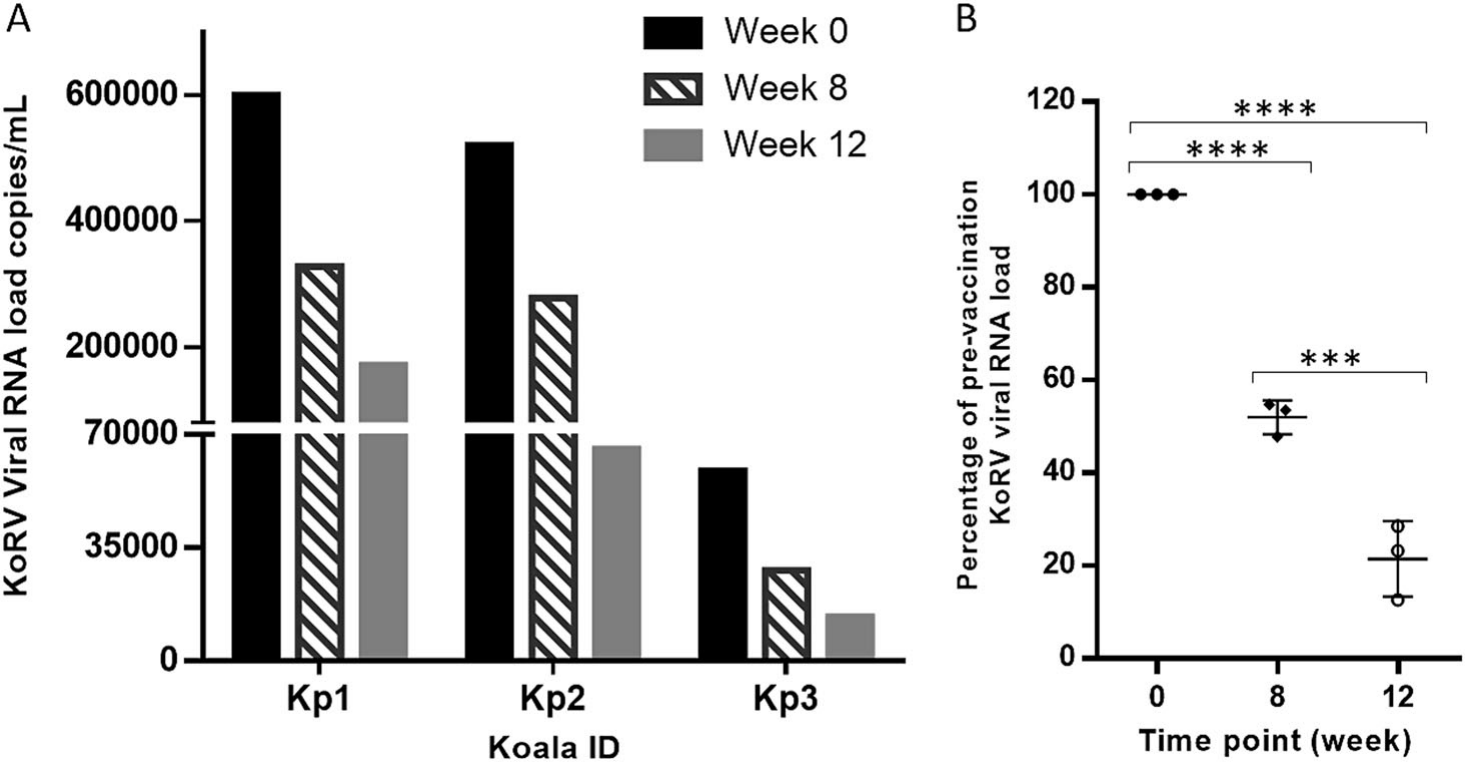
Change in KoRV viral RNA load following vaccination in KoRV-infected koalas as measured by RT-qPCR. **a** Absolute KoRV viral load in each KoRV-positive koala over the trial period. **b** KoRV viral load presented as percentage of pre-vaccination in KoRV-positive koalas (*n* = 3) over time (mean ± SEM are represented by horizontal lines and error bars). The level of significance was measured as ***p = 0.0003 and ****p < 0.0001 using Student’s *T* test (*p* ≤ 0.05)

To determine the efficacy of our vaccine in reducing KoRV viral load in vivo, KoRV viral RNA load present in plasma at weeks 0, 8, and 12 post-vaccination was measured using a KoRV envelope gene-specific one-step RT-qPCR assay. Reduction in viral load was observed in all three KoRV-positive vaccinated koalas between weeks 0 and 8 and this further reduced by week 12 (Fig. 5a). Overall, the average viral load of the vaccinated KoRV-infected koalas at week 8 represents a 48% reduction while week 12 represents a 79% reduction in the viral load compared to levels at week 0. To more accurately analyze the reduction in viral load among the three koalas, KoRV viral load was normalized to a fixed value, 100, and the mean at each time point was compared using a one-way ANOVA. The analysis showed a significant reduction in KoRV viral load at each time point post-vaccination (Fig. 5b). The level of significance was measured as *p = 0.0003 and **p < 0.0001 using Student’s *T* test (*p* ≤ 0.05).

### Characterization of individual epitope response in vaccinated animals

**Fig. 6:**
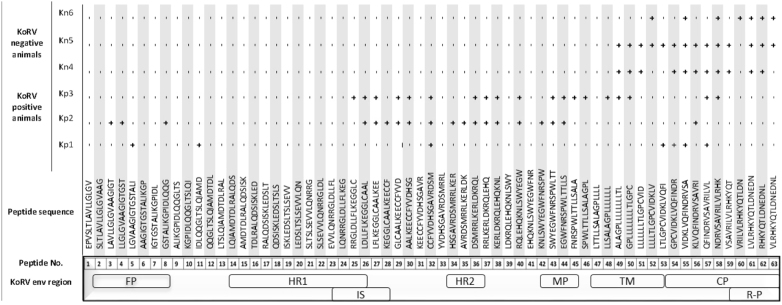
List of epitopes across the p15E subunit of the KoRV env protein recognized post-vaccination by KoRV-positive and KoRV-negative koalas + Recognized epitopes; - unrecognized epitopes; FP fusion peptide; HR1 heptad repeat 1; IS immunosuppressive domain, HR2 heptad repeat 2, MP membrane proximal external region, TM transmembrane region, CP cytoplasmic tail, R-P R-peptide.

To investigate what linear epitopes are recognized post-vaccination, we designed 63 overlapping 15mer peptides with three amino acids offset to span the full length of the ecto- and endo-domains of the p15E subunit of KoRV envelope protein. Each biotinylated peptide contained a hydrophilic tetrapeptide, SGSG, between the biotin and sequence of interest. Peptides 1–46 and peptides 27–63 represent the ecto-domain and endo-domain, respectively, of the KoRV envelope protein p15E subunit. Using a newly developed peptide ELISA, the response of each pre-vaccination sample was compared against its 8 weeks post-vaccination sample. For the KoRV-negative koalas evaluated, vaccination resulted in similar responses pre- and post-vaccination for peptides 1–48, indicating that these epitopes were not recognized. Peptides 49–63, representing the endo-domain, however, showed an increased response post-vaccination. When plasma samples from KoRV-positive animals were evaluated against the same set of peptides, the antibody profiles were markedly different from that of the KoRV-negative animals. While six epitopes present in the endo-domain sequence, also recognized by the KoRV-negative koalas, showed increased responses, more epitopes within the ecto-domain region of p15E subunit showed increased response post-vaccination in KoRV-infected koalas (Fig. 6).

## Discussion

Two main infectious diseases, *Chlamydia* and KoRV, including a potential interaction between them, pose a significant threat to the long-term survival of the koala. KoRV-related lymphomas have been reported in several captive populations and, while not as prevalent, also occur in wild koala populations.^6,7^ KoRV has also been shown to be associated with chlamydial disease in wild koalas.^10^ In addition to losses in wild koalas, both diseases also cause significant morbidity and mortality in captive animals. While quarantine and antiretroviral drug treatment are possible control measures, they are generally impractical in a wild animal such as the koala, leaving vaccination as the only realistic option. In this study, we examined the effect of a recombinant envelope protein-based anti-KoRV vaccine in koalas, either naturally infected with KoRV-A or KoRV free. Our results show that vaccination can be successful for both KoRV-negative and KoRV-positive koalas. All vaccinated animals produced significant levels of circulating anti-KoRV antibodies. We showed that these antibodies had a neutralizing effect in an in vitro assay, but more importantly, we showed that vaccination had a therapeutic effect on koalas infected with KoRV by significantly reducing the circulating viral load. We also identified B-cell epitopes that were differentially recognized in KoRV-infected versus KoRV-free koalas following vaccination.

Using the same recombinant protein that was used as the vaccine immunogen, rEnv, we successfully developed an ELISA to measure the KoRV-specific serum IgG response following vaccination. We found that our vaccine formulation induced a significant antibody response in all six koalas. This finding is consistent with a small preliminary trial^22^ that was conducted in Queensland koalas with endogenous KoRV-A infection where vaccination was shown to induce an immune response. In our current trial, we purposely chose South Australian animals that were infected with what is assumed to be exogenous KoRV, as well as those that were KoRV negative, to compare the immune responses to vaccination. We found that the antibody levels 3 months post-vaccination remained significantly higher than pre-vaccination levels and the average response was comparable in both KoRV-positive and -negative animals. This finding demonstrates that the recombinant protein vaccine antigen is effective in stimulating immune response in naturally infected koalas.

A key finding of our study was that all the vaccinated koalas, both KoRV free as well as those exogenously infected with KoRV, produced vaccine-induced antibodies that were able to neutralize KoRV in an in vitro assay. We also found that the levels of neutralizing antibodies produced following vaccination were similar in both KoRV-negative and KoRV-infected koalas. This is strongly encouraging for KoRV vaccine development. The p15E subunit of the envelope protein has been shown in other studies to induce the production of neutralizing antibodies following vaccination against other retroviruses such as FeLV, FIV, PERVs, HIV-1, SRV-1,^23–27^ with the KoRV p15E subunit, in particular, shown to induce neutralizing antibodies in rats.^28^ As this is an encouraging finding, it will be interesting to determine if the antibodies produced against this subunit represent broadly neutralising antibodies. KoRV, like other retroviruses, has been shown to possess sequence type variations in the envelope protein,^29^ and these variations have been used for classification purposes.^4,30^ Although we tested the ability of the post-vaccination serum to neutralize KoRV-A variant only, the possibility of the serum to neutralize other KoRV variants must also be investigated.

Following the promising antibody response to vaccination, particularly the neutralizing ability, we characterized the specificity of the antibodies to epitopes within the env protein using PepScan methodology. The protein contains epitopes which are responsible for the production of neutralizing antibodies in FeLV, FIV, PERVs, HIV-1, and SRV-1. The p15E region contains hydrophilic domains which provide stronger water solubility and stronger polarity. Both properties make the recombinant protein a suitable vaccine antigen. We found two distinct epitope profiles in the p15E subunit region following vaccination. Following vaccination, KoRV-negative koalas produced antibodies specific to epitopes only in the endo-domain region of the p15E subunit of the env protein. While previous studies have targeted the ecto-domain region of the transmembrane protein of retroviruses for the induction of neutralizing antibodies,^31^ this study showed that epitopes on the endo-domain region of the transmembrane protein may also induce production of neutralizing antibodies. Koalas naturally infected with KoRV, on the other hand, produced antibodies against epitopes mainly in the ecto-domain but also a few epitopes in the endo-domain of the transmembrane protein. It was interesting to find that koalas differentially recognize B-cell epitopes following KoRV vaccination based on previous exposure to KoRV, and this finding could prove to be very important in future design of KoRV vaccines by targeting specific peptides for KoRV-infected or KoRV-negative animals.

Another important finding in this study was a steady reduction in KoRV viral RNA load over the trial period in all three KoRV-infected koalas following vaccination. While the production of antibodies, including neutralizing antibodies, following vaccination is promising, the most critical requirement of a vaccine is whether it can reduce the viral load in infected animals. Viral loads in infected animals dropped by an average of 48% by 8 weeks post-vaccination and by 79% by 12 weeks post-vaccination. We consider this reduction to be quite significant considering the relatively short time frame (12 weeks) post-vaccination. Although, we could not measure the responses at longer times due to ethical limitations of holding the koalas for longer periods, we expect that this trend in viral load reduction levels would continue. This is highly encouraging for future therapeutic KoRV vaccine development.

Viral load has been shown to correlate with disease progression in other retroviruses such as FIV, HIV-1, and SIV,^32–34^ and transmission in HIV.^35^ While there has not yet been any direct study on koala disease outcome in response to manipulation of KoRV viral load, a related study does exist.^7^ It was shown that KoRV viral loads were significantly higher in koalas with leukemia or lymphoma, relative to healthy animals. Therefore, the assumption is that reducing viral loads should reduce the associated disease progression. The reduction in viral load reported in this study might be important in delaying KoRV-associated disease progression as well as reducing the KoRV transmission rate, since exogenous transmission is presumably dependent on viral load. Therapeutic vaccines represent a promising approach in the effort to enhance immunogenicity, reduce bacterial and viral load in both human and animal infections, and reduce pathogen associated morbidity and mortality.^20,21,36–39^ The importance of a therapeutic vaccine, especially in a single dose format, becomes even more apparent in the case of wild koalas where antiretrovirals do not represent a viable option in treating KoRV infections. The results of this study suggest that the therapeutic effect seen in this trial population, though limited in size, should be applicable to a larger population of koalas, thus making our vaccine suitable for use in the wild. While our results are promising, we recognize that further work is needed to refine the vaccine into a single dosage to suit wild koalas. Nevertheless, the vaccine could potentially be used in its current form in koalas which are KoRV negative or exogenously infected with KoRV-A. These koalas are an important subgroup of animals to protect against KoRV with the aim of preventing or delaying further KoRV transmission, associated disease, and/or endogenization. Such koalas exist in South Australia and Victoria.

The long-term survival of the koala is under threat from many human causes (such as habitat destruction) as well as infectious disease, chiefly *Chlamydia* and KoRV. KoRV is spreading through the Australian koala population, apparently from north to south, with 100% of northern koalas infected (endogenously) but only around 50% of southern animals currently infected (exogenously). A vaccine that could be effective, even in just exogenously infected koalas, would be a major advance. Our results demonstrate very promising progress in the development of an effective anti-KoRV vaccine in both naïve as well as exogenously infected animals. The next stage will be to evaluate this vaccine in endogenously infected koalas. Vaccines against other retroviruses, such as FeLV, have been shown to be successful and if an equally successful vaccine can be developed for koalas, then this should have a significant impact on saving the species.

## Materials and methods

### Preparation of recombinant KoRV Env protein

The Env protein of KoRV, based on the multiple alignment of available KoRV sequences in GenBank (http://www.ncbi.nlm.nih.gov/), was optimized for expression in *Escherichia coli* (Fig. 1a and b). The optimized gene was synthesized and cloned into pGEX-4T1 with Bam HI and Sal I restriction sites at the 5′ and 3′ ends of the gene, respectively. The correct sequence of the construct was confirmed by sequencing at both forward and reverse directions (AGRF, Adelaide). The expression vector carried Glutathione S-transferases (GST) for further affinity purification. The recombinant vector was transformed into *E. coli*, BL21 cells. For the expression of the recombinant protein, a fresh subculture of the transformed cells was cultured in a rich Luria-Bertani Medium (10 g Tryptone, 10 g NaCl, 5 g yeast extract, and 0.1% D-glucose per liter of distilled water) containing 30 mg/ml of filtered sterile Ampicillin, then induced by adding 0.3 mM isopropyl b-D-1-thiogalactopyranoside (IPTG) (Sigma, St Louis, MO, USA) with subsequent incubation for 3 h at 250 rpm at 37 °C. The culture suspension was centrifuged at 5000×*g* for 20 min at 4 °C in (Sorvall RC 5B centrifuge). The supernatant was discarded, and the cells were resuspended in cold lysis buffer (20 mM Tris HCl pH 8.0, 300 mM NaCl, 1 mM 2-mercaptoethanol, and 5% Triton X-100). To lyse the cells, a final concentration of 60 µg/ml lysozyme and 200 µg/ml DNase I were added to the cell suspension and incubate on ice for 20 min till the liquid turns to a non-viscous and cleared lysate. The suspension was centrifuged at 8000×*g* at 4 °C for 10 min. The supernatant was filtered through 0.22 µM syringe filters and used for affinity purification chromatography. The obtained cell lysate containing GST-Env-Trans protein was loaded onto a GST column (GE Healthcare, life sciences) and washed to remove all other impurities. The GST-ENV-Trans protein eluted with column buffer containing 20 mM Glutathione (Sigma, St Louis, MO, USA). The purified protein was desalted and concentrated by ultrafiltration in Vivaspin size exclusion centrifugal membrane tube with cut-off 20,000 Dalton (Sartorius Stedim Biotech, Goettingen Germany). Protein concentration of the GST-Env-Trans, hereafter denoted by rEnv, was measured using NanoDrop, adjusted at 100 mg/ml and stored in aliquots at −70 °C. To analyze the purified protein, 10 µl of the protein was run in a 12% discontinues odium dodecyl sulfate polyacrylamide gel electrophoresis.

### Adjuvant

The adjuvant used was a three-compound mixture that consists of polyphosphazine (PCEP), host defense peptide 1002, and poly I:C (Vaccine and Infectious Disease Organization, Saskatchewan, Canada).^40^ Each dose of vaccine was prepared as per Khan et al.^40^ with sterile phosphate buffered saline (PBS) to contain 50 µg of rEnv, with 250 µg each of PCEP, poly I:C, and 500 µg of peptide 1002.

### Immunization and sample collection

Six healthy koalas, as assessed by veterinary staff and blood biochemical parameters from the Mount Lofty population in South Australia, were housed temporarily at the Adelaide Koala and Wildlife Hospital (Plympton, South Australia) whilst awaiting release following veterinary treatment for minor conditions and used for this study. The koalas were assigned into two groups based on their KoRV infection PCR status: (a) KoRV positive or (b) KoRV negative. Animals in both groups were vaccinated subcutaneously on day 0 and received a booster shot at 4 weeks. Four milliliters of whole blood was collected on day 0 (prior to vaccination), and at weeks 8 and 12 post-vaccination. Blood samples beyond 12 weeks post-vaccination could not be obtained due to regulations related to holding koalas in captivity for longer periods; 500 µl of whole blood was stored at −20 °C for genomic DNA extraction for KoRV infection status. Serum and plasma samples were obtained and 100 µl of plasma was placed into RNALater at −20 °C for quantification of KoRV viral RNA load. All animal work was approved by University of the Sunshine Coast Animal Ethics Committee (AN/A/16/106), University of Adelaide Animal Ethics Committee (S-2016-053) and the South Australian Government (Department of Environment, Water and Natural Resources scientific purposes permit, E26513-1).

### KoRV genotyping and estimation of proviral copy number

All koalas were screened for KoRV infection using a genomic DNA conventional PCR assay and quantitative PCR of viral RNA extracted from plasma as per ref. ^10^ The additional qPCR step was conducted to confirm that any koala designated as being KoRV positive is not merely carrying replication defective KoRV as it has been suggested that some South Australian koalas may test positive for a target proviral gene but not express the same at the transcriptome level.^41^ Briefly, PCR primers were designed to amplify part of the highly conserved polymerase (pol) gene to confirm the presence or absence of KoRV infection, while the variable region A and transmembrane p15e domain of the KoRV envelope (env) gene were used to differentiate between KoRV-A and KoRV-B virus types.^10^ To confirm the specificity of our KoRV-A specific PCR assay, the PCR products from the three positive animals were sequenced (Macrogen Inc.) and matched to the published KoRV-A sequence (Genbank accession AF151794). Proviral copy number was quantified as per ref. ^2^

### Total KoRV viral RNA PCR and quantification

Viral RNA was extracted from the plasma stored in RNALater using the Qiagen viral RNA mini kit (Qiagen). Contaminating DNA was removed with Amplification Grade DNase I (AMPD1; Sigma). Viral RNA load was thereafter quantified using QuantiTect SYBR Green RT-PCR Kit (Qiagen). For quantification of viral RNA, standards of known concentration (10^8^ to 10^1^) of KoRV env gene plasmid prepared by Integrated DNA technologies (Coralville, Iowa, USA), were prepared for each run and the results were normalized against the standard curve. PCR reactions were carried out using CFX 96 Touch System (Bio-Rad, Australia) under the following conditions: env; 50 °C for 30 min, 95 °C for 15 min (1 cycle), 94 °C for 15 s, 54 °C for 30 s, 72 °C for 30 s (50 cycles). All procedures were carried out following the manufacturers’ instructions.

### KoRV-specific IgG response ELISA

To characterize accurately anti-KoRV antibodies following vaccination, we removed any serum antibodies that may be directed against the GST component of the rEnv used for both vaccination and immunoassay. We coated 96-well ELISA plates (Clear Flat-Bottom Immuno Nonsterile; ThermoFisher Scientific, Aus) with 1 µg GST and incubated at room temperature for 2 h. The plate was thereafter washed three times with PBS containing 0.05% Tween-20, and incubated with serum samples at 4 °C overnight. Serum samples were collected afterwards and used in the ELISA described below; 96-well ELISA plates (Clear Flat-Bottom Immuno Nonsterile; ThermoFisher Scientific, Aus) were coated overnight at 4 °C with 1 µg rEnv in PBS and blocked for 1 h at 37 °C with 5% skim milk in PBS containing 0.05% Tween-20 (smPBST) on a shaker. Serum samples were serially diluted two-fold in smPBST, with a starting dilution of 1:100, added to wells and incubated on a shaker at 37 °C for 1 h. Sheep anti-koala IgG diluted 1:5000 in smPBST was added to the plates and incubated on a shaker for 1 h at 37 °C. Thereafter donkey anti-sheep HRP IgG (In Vitro Technologies, Aus) diluted 1:1000 in smPBST was added and incubated for a further 1 h at 37 °C on a shaker. The plate was washed three times with PBS containing 0.05% Tween-20 (PBST) after each step; 100 µl of Tetramethylbenzidine (TMB) Liquid Substrate (Sigma-Aldrich) was added and the reaction was stopped after 3 min with 100 µl of 1 m H_2_SO_4_. Optical density was read at 450 nm (EnSpire Multimode Plate Reader, PerkinElmer, Australia) and end-point titers thereafter calculated (GraphPad Prism 6.01).

### Anti-KoRV MPER peptide IgG ELISA

A peptide designated as KoRV MPER and containing 31 amino acids (LKERLDKRQL EHQKNLSWYE GWFNRSPWLT T), representing the MPER of KoRV envelope protein (Fig. 1b), designed by our group and synthesized (95.4% purity) by VCPBIO (Shenzhen, China) was used to coat 96-well ELISA plates at a concentration of 1 µg in bicarbonate buffer for 1 h at 37 °C on a shaker. The plate was washed three times in PBST and thereafter blocked with smPBST. Serially diluted sera were added and incubated at 37 °C on a shaker for 1 h. All other reactions proceeded as per KoRV-specific IgG response ELISA described above.

### Design of biotinylated KoRV p15E peptide library

The p15E sequence of KoRV envelope protein (464–659 AA) was used to design 63 overlapping 15mer peptides with 3 amino acids offset. We designed the peptides to span both the full length of ecto- and endo-domains of the p15E subunit of KoRV envelope protein (Fig. 1c). Each biotinylated peptide (constructed by Mimotopes; Melbourne, Australia) contained a hydrophilic tetrapeptide, SGSG between the biotin and sequence of interest.

### KoRV p15E epitope mapping ELISA

Wells of streptavidin-coated 96-well plate (Mimotopes, Aus) were coated with individual peptide at a concentration of 2 µg/well in PBS containing 0.1% Tween-20 (PBST) and incubated for 2 h at room temperature on a shaker. Then the plate was washed four times with PBST and coated for 4 °C overnight with plasma samples diluted 1:50 in PBST. Post-incubation, the plate was washed four times in PBST and a secondary antibody, sheep anti-koala IgG, was added at a dilution of 1:5000 in PBST and incubated for 1 h at room temperature on a shaker, and thereafter washed four times in PBST. A detecting antibody, donkey anti-sheep HRP IgG (In Vitro Technologies, Aus), diluted 1:1000 in PBS was added and incubated for a further 1 h at room temperature on a shaker. The plate was then washed four times with PBST and a further two times with PBS to remove traces of tween-20; 50 µl of 1-Step™ ABTS Substrate Solution (2,2′-Azinobis [3-ethylbenzothiazoline-6-sulfonic acid]-diammonium salt, ThermoFisher Scientific, Aus) was added and incubated at room temperature for 10 min. Color development was measured at an optical density of 405 nm wavelength (EnSpire Multimode Plate Reader, PerkinElmer, Australia). Data analysis was performed as per ref. ^42^

### KoRV in vitro neutralization assay

Prior to setting up KoRV in-vitro neutralization assay, we isolated KoRV virus stock. Briefly, concanavalin A-stimulated PBMC from KoRV-A-infected Queensland koala was cocultured with HEp2 cell line. Afterwards, filtered supernatant was collected and added to fresh HEp2 culture. Filtered supernatant from infected HEp2 cells were passaged five times. To confirm infectivity, filtered supernatant from infected HEp2 cells was added to HEK 293T cell line and passaged six times. Filtered supernatant from both HEp2 and 293T cultures were tested by PCR for presence of KoRV and designated as KoRV virus stock. To confirm the specificity of isolated KoRV, the PCR products were sequenced (Macrogen Inc.) and matched to the published KoRV sequences. KoRV in vitro neutralization assay was performed as per ref. ^43^ Briefly, serum samples decomplemented by heating in a water bath at 56 °C for 30 min, and diluted at a ratio of 1:20 in cell culture medium. KoRV virus stock was added, at a ratio of 1:2, to serum samples and incubated for 30 min at 37 °C under 5% CO_2_. Virus stock that gave a Cq value of 25–27 was used for infection. Culture medium was removed from HEK 293T cells (ATCC, CRL-11268) seeded in 96 well plates (Greiner bio one; Interpath, West Heidelberg, Australia) and grown until they reached 50–60% confluence, and replaced with serum samples. The culture was incubated for 65 h at 37 °C under 5% CO_2_. Post-incubation, the cells were harvested, genomic DNA was extracted using QiaAmp DNA mini kit (Qiagen) following the manufacturer’s instructions, and analyzed for KoRV proviral integration. Total proviral load was then quantified as per ref. ^10^ Neutralization was determined by comparing the proviral load of infected cells in the presence of post-immunized serum samples to pre-immunized samples.

### Data availability

The data that support the findings of this study are available from the authors on reasonable request.
